# Maternal Iron Levels and Association With Gestational Diabetes: A Systematic Review and Meta-Analysis

**DOI:** 10.1155/jnme/1772306

**Published:** 2025-04-24

**Authors:** Jinguang Wang, Zhen-Yu Chen, Jian Shen, Huan-Juan Ni, Jingli Sun

**Affiliations:** Department of Obstetrics and Gynecology, General Hospital of Northern Theater Command, No. 83, Wenhua Road, Shenhe District, Shenyang 110016, China

**Keywords:** ferritin, gestational diabetes mellitus, iron, meta-analysis, transferrin

## Abstract

**Background:** This systematic review aimed to assess the association of iron level with gestational diabetes mellitus (GDM) risk.

**Methods:** The relevant articles published between January 1, 1995 and January 17, 2023 were identified through a systematic literature search. This study used random effects to summarize the relative risks (RRs) 95% confidence intervals (CIs) of GDM risk and standardized mean differences. This study investigated the association of ferritin exposure with GDM combined with dose–response analysis and explored both linear and nonlinear trends.

**Results:** This meta-analysis selected 30 studies with serum ferritin (SF), 18 studies with serum iron (SI), 4 studies with serum transferrin receptor (sTfR), 5 studies with total iron binding capacity (TIBC), and 4 studies with transferrin (TRF). The summarized RRs comparing persons with the highest concentration categories of SF with the lowest concentration categories of SF with an unadjusted odds ratio were 2.05 (1.67–2.53; *I*^2^ = 62.8%, *p* < 0.001, *z* = 6.76, *p* < 0.001) and with an adjusted odds ratio were 1.82 (1.54–2.14; *I*^2^ = 12.9%, *p*=0.312, *z* = 7.21, *p* < 0.001). Linear dose–response showed that an increase in SF of 5 μg/L increased the risk of GDM by 2.66% (1.026 [95% CI: 1.017, 1.036], *n* = 5). The nonlinear dose–response relationship also indicates that the increased SF is consistently associated with an increasing risk of GDM.

**Conclusion:** High ferritin, high iron levels, and low TIBC are associated with an increased risk of GDM.

## 1. Introduction

Gestational diabetes mellitus (GDM) is one of the most prevalent complications during pregnancy and has been defined as any degree of glucose intolerance with onset or first recognition during pregnancy [1]. It is diagnosed in the second or third trimester of pregnancy and is not overt diabetes before gestation [2], affecting 1%–14% of all pregnancies worldwide each year [3]. According to the recent International Diabetes Federation (IDF) estimates, GDM affects approximately 16.7% of births annually [4, 5]. GDM is associated with both short-term and long-term adverse perinatal outcomes for offspring, as well as an increased risk of maternal complications [6]. Abortion, premature birth, postpartum hemorrhage, shoulder dystocia, perineal lacerations, and birth complications in the infant such as fetal growth restriction, macrosomia, increased risk of developing type 2 diabetes, and cardiovascular diseases in maternal and newborn, neonatal hypoglycemia, neonatal hyperbilirubinemia, congenital malformations, and intrauterine fetal death are the maternal complications of women with GDM [7, 8].

The physiological mechanism underlying GDM remains unclear. Some studies have shown that it is similar to type 2 diabetes in that the main pathogenesis of GDM is likely to be caused by a decreased insulin secretion and insulin resistance during pregnancy, oxidative stress and inflammatory response, insulin receptor mutations, insulin signal transduction defects, pancreatic β-cell dysfunction that may cause insulin resistance, and the iron overload that may damage pancreatic β-cell, which affects glucose metabolism [9–11]. Iron overload from chronic primary conditions and transfusions, as well as a prolonged intake of supplements with high iron content, can lead to systemic iron overload, potentially causing an increased accumulation of unabsorbed iron in the intestine. The effects of excess iron can be systemic or localized. Accumulated iron may lead to the increased production of oxidative stress substances in the intestine, which could exacerbate inflammation reactions and disrupt normal insulin secretion. This accumulation of iron might also promote the production of more oxidative stress substances in the intestine, potentially intensifying inflammation reactions, interfering with normal insulin secretion, and subsequently contributing to the development of diabetes [12]. Pregnant women are at a risk of ferritin deficiency [13]. Iron deficiency is associated with adverse pregnancy outcomes, such as increased maternal illness, low birth weight, and intrauterine growth restriction [14, 15]. However, excess iron is associated with adverse pregnancy outcomes, including GDM [16]. In recent years, some studies have confirmed that excess iron is associated with GDM, but others have the opposite findings [17–39].

While the relationship between iron levels and GDM has been widely investigated, this study enhances the existing evidence by incorporating a dose–response meta-analysis to explore both linear and nonlinear trends. By synthesizing data from multiple studies, this research provides a comprehensive evaluation of the potential impact of ferritin levels on GDM risk. Despite the limited novelty, the findings hold clinical significance by offering insights that may aid in risk assessment and early intervention strategies for GDM. Therefore, this study aimed to investigate the impact of iron levels on GDM risks.

## 2. Materials and Methods

### 2.1. Retrieval of Literature

This study was conducted following the criteria of Meta-analysis of Observational Studies in Epidemiology (MOOSE) [17]. Firstly, this study involved the identification of relevant literature examining the association of iron status with the risk of GDM. This search retrieved several articles from different databases, including 643 articles form Embase and 132 from PubMed up to January 17, 2024. Although this study primarily searched Embase and PubMed, other major databases such as Web of Science and Scopus were not included. This limitation may have led to the omission of certain relevant studies. However, to enhance comprehensiveness, reference lists of included studies were manually screened to identify additional eligible articles, reducing the likelihood of missing key research findings. The literature search utilized the following keywords: “Ferritin,” “Ferritins,” “Isoferritin,” “Isoferritin, Basic,” or “Iron” in combination with “Diabetes, Gestational,” or “Diabetes, Pregnancy-induced” or “Pregnancy-induced Diabetes” or “Gestational Diabetes” or “Diabetes Mellitus, Gestational” or “Gestational Diabetes Mellitus.” Additionally, references and citations were carefully reviewed to avoid duplication of studies. Only 39 studies were included that dealt with humans and were published in the English language.

### 2.2. Criteria for Inclusion or Exclusion of Data

This study involved the following criteria set for the inclusion of a study: (1) studies involving the human; (2) studies that investigated the relationship of body's iron level (ferritin, transferritin, iron, sTtR, and total iron binding capacity (TIBC)) with GDM risk; (3) prospective cohort, case–control studies; and (4) studies narrating the full-text review relative risks (RRs) or standardized mean difference (SMD) or standard deviation (SD) (or median and ranges/interquartile ranges) or unadjusted and/or adjusted odds ratio (OR) and 95% CI or the binary outcomes in pregnant women with vs. without GDM from reported data.

Exclusion criteria were (1) studies that involved animals; (2) review articles, editorials, meetings, abstracts, commentaries, and unpublished studies; and (3) studies with inadequate (e.g., considerations such as the quantity and quality of data, methodological soundness, consistency of data, and the capability for statistical analysis of results) or non-original data.

### 2.3. Study Selection Process

All articles were estimated by two independent reviewers (JGW and ZYC) by using the inclusion and exclusion criteria described. When two reviewers disagreed, the article was reviewed and agreement was reached by consensus with a third reviewer (JLS). To minimize bias or unfairness in the peer review process, the third reviewer remained unaware of the specific opinions or decisions of the other two reviewers before reaching a consensus.

### 2.4. Data Extraction and Quality Assessment

From each included article, the following data were extracted in this meta-analysis: basic information (title, author, publication year), study characteristics (name of the article, study design, country), participant characteristics (sample size, GDM diagnosis criteria, assay type, number of GDM patients and controls, age, gestational age that first to third trimesters), reported iron indices (assessment of body iron stores), and outcome index. Risk estimates and 95% CIs of GDM, any covariates adjusted in the multivariate analysis, mean, and SD (or median and ranges/interquartile ranges) of iron index markers are the characteristics of outcome index.

The quality analysis of each included study was done by two authors (JGW and ZYC), independently, according to the Newcastle–Ottawa Quality Assessment Scale (NOS), and discrepancies were resolved by discussions by a comparison of scoring rationale, standardized interpretation discussion, repeated evaluation, and revision. If the two reviewers still cannot reach a consensus after the discussion, consider introducing a third reviewer as an arbitrator. The NOS checklist contains three parts: (1) the selected population; (2) the comparability of study groups; and (3) the assessment of exposure or outcomes for case–control or cohort studies. In this meta-analysis, the included studies were assigned a score from 0 to 9. Studies with NOS scores above 6 were classified as high quality.

### 2.5. Statistical Analysis

Analysis was conducted by STATA statistical software version 12.0. The SMDs were pooled for continuous variables and a pooled ORs and RRs for blood levels of ferritin, iron, TIBC, transferrin, and transferrin receptor by random effect models or fixed-effect model [18, 19]. The extent of heterogeneity was measured using the *I*^2^ statistic and *I*^2^ values of 25%, 50%, and 75%, respectively, which was considered as low, medium, and high heterogeneities [20]. The *I*^2^ > 50% indicated a significant heterogeneity between studies, and the random effects model was selected. Alternatively, the fixed-effect model was used when there was no significant heterogeneity across the studies. This study explored heterogeneity in sensitivity analyses by stratifying based on study size, continent, age, and study design and recalculated the pooled effect by deleting the low-quality studies (NOS score below 6 stars). Publication bias was assessed by Begg's test and Egger's test. The possibility of publication bias in the included studies is low when *p* > 0.05.

This study conducted a meta-analysis of a dose and its response utilizing the Greenland and Longneck technique, supposing a quadratic association between the continuous exposure [21]. The alternative two-stage approach evaluated the same model independently of each research.

The iron index and risk variance calculations included a minimum of three quantitative exposure categories. When the median or mean exposure levels for this category were not provided, the categorical midpoint was used. If the highest or lowest category was unbounded, the midpoint of the category was approximated by assuming the width of the category to be equivalent to that of the neighboring category. Consequently, the dose–response meta-analysis will be confined to the subset of the remaining four trials [28, 40–42]. The dose–response relationship requires centralized data. The nonlinear dose–response meta-analysis uses limited cubic splines as the connecting function. In the Stata software, the mkspline function is used for the fitting of restricted cubic splines. The segmentation of intervals is done using percentiles (5%, 35%, 65%, and 95%), and the _pctile function is employed to generate four percentiles for dose, resulting in four regression splines. To ascertain whether the combined dose–response relationships in the meta-analysis are nonlinear, one needs to examine the statistical test results, specifically the *p*-value. When the *p*-value is less than 0.05, it indicates a nonlinear relationship. If the *p*-value is close to 0.05 (0.04–0.06), both linear and nonlinear relationships are considered. A *p*-value greater than 0.05 suggests a linear relationship. This approach aids in understanding the complex relationship between dose and response and assessing the statistical significance of these relationships in meta-analysis [23].

## 3. Results

### 3.1. Literature Selection

**Figure 1:**
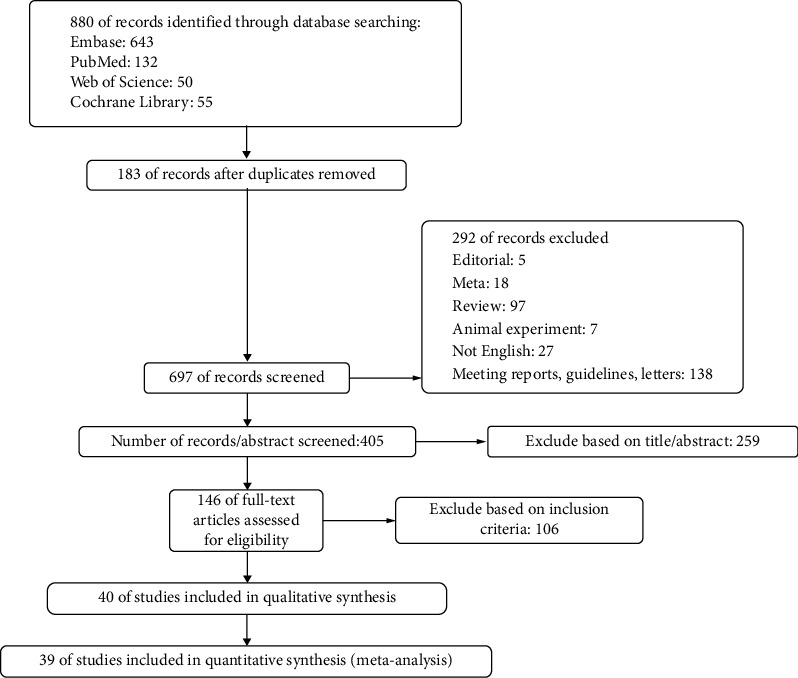
Selection process of studies.

The literature search through four databases, EMBASE, PubMed, Web of Science, and Cochrane Library, identified 880 potentially relevant studies. After removing duplicates, 697 articles were identified. Of these articles, 292 were excluded by exclusion criteria. Of the rest 405 articles, this study screened titles and abstracts and then evaluated the full texts. Two-hundred and fifty nine and 106 were excluded by the pre-specified criteria, respectively. Finally, this study reviewed 39 articles involving a total of 22,449 women (4603 GDM patients and 17,846 non-GDM pregnant women).  Figure 1 represents the selection layout of this study. Finally, the Preferred Reporting Items for Systematic Reviews and Meta-analysis (PRISMA) guidelines were applied to conduct this meta-analysis.

### 3.2. Characteristics of the Included Studies

A detailed list of information on the selected studies is summarized in Table 1. A total of 39 eligible studies were identified in the quantitative meta-analysis [24–62]. The studies were published from 1997 to 2022, and the ages of pregnant women ranged from 18 to 45. Out of the 39 included studies, 16 were cohort studies and 23 were case–control studies. Thirty studies examined the association between serum ferritin (SF) levels [24–30, 32–47, 51, 54, 55, 57, 59–61] and the risk of GDM; 18 studies assessed the relationship between serum iron (SI) [27, 31, 32, 34, 35, 37, 39, 43, 45–49, 51, 52, 56, 58, 63] and GDM; five studies with TIBC [26, 30, 33, 38, 47], four studies with serum transferrin receptor (sTfR) [24, 27, 28, 59], and four studies with transferrin [36, 37, 47, 48] were selected for the meta-analyses.

The Newcastle–Ottawa qualitative assessment median bias risk score of prospective cohort studies and case–control studies was 8.0 (range: 6–9) and 6.95 (range: 6–8), respectively (Table 2). All studies had six or more stars on the modified NOS.

### 3.3. Serum Ferritin

There were 30 studies on the comparison of SF levels between the GDM group and non-GDM group [16–22, 24–30, 32–47, 51, 54, 55, 57, 59–61, 63].

#### 3.3.1. Mean Ferritin Concentration

**Figure 2:**
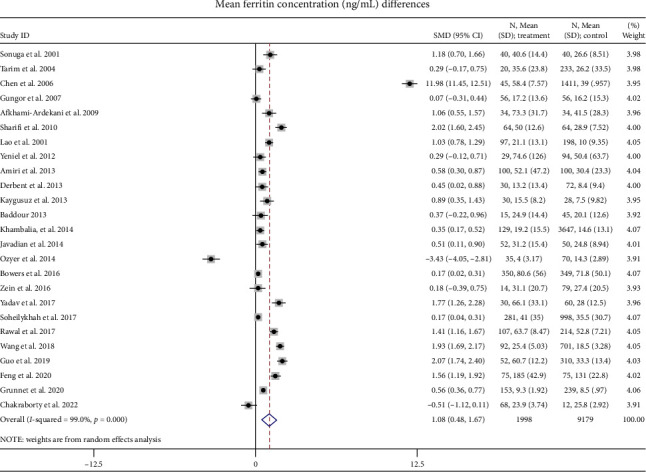
Mean ferritin concentration (ng/mL) differences.

Twenty-five articles were added to this meta-analysis of the mean ± SD of SF levels in GDM and non-GDM groups. The findings revealed a statistical heterogeneity between the study results (*I*^2^ = 99.0%, *p* < 0.001), and a random-effects model was chosen for meta-analysis. The outcomes exhibited that the SF level in the GDM group was significantly higher by 1.08 ng/mL (95% CI: 0.48 to 1.67, *p* < 0.001) compared with the non-GDM group (Figure 2). This statement suggests that in the group with GDM, the SF level was 1.08 ng/mL higher compared to the non-GDM group. Furthermore, the difference in these levels was found to be statistically significant (*p* < 0.05), indicating that the observed variation is not likely due to random chance.

#### 3.3.2. Ferritin Concentration

This review included a total of 14 articles, which focused on the relationship between SF levels and the risk of GDM. Based on the SF levels of the included studies, the levels were divided into a high-level SF group and a low-level SF group. Meta-analysis was performed based on the unadjusted and adjusted parameters.

**Figure 3:**
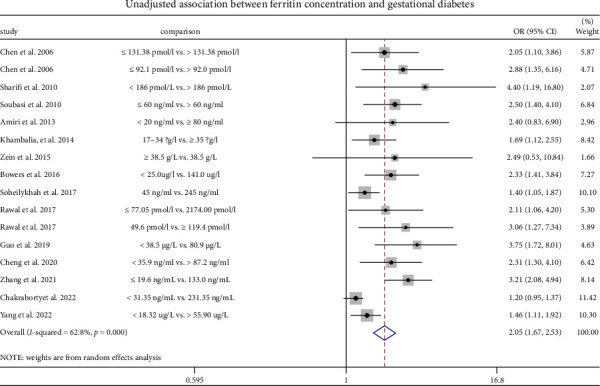
A representation of the unadjusted relationship of ferritin concentration with GDM.

**Figure 4:**
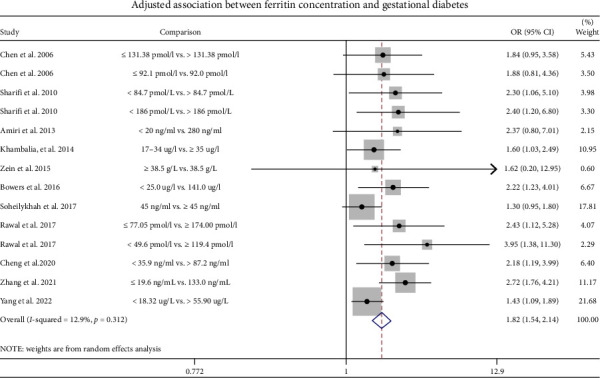
A representation of the adjusted relationship of ferritin concentration with GDM.

The elevated levels of ferritin were linked with GDM with higher levels of SF. An unadjusted OR was 2.05 (95% CI: 1.67–2.53; *I*^2^ = 62.8%, *p* < 0.001) (Figure 3), while the adjusted OR was 1.82 (95% CI: 1.54–2.14; *I*^2^ = 12.9%, *p* < 0.001), compared with those with lower ferritin levels (Figure 4).

### 3.4. Serum Iron

**Figure 5:**
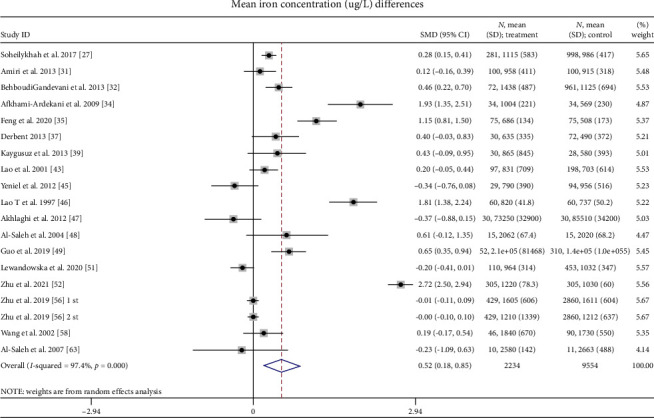
Mean iron concentration (μg/L) differences.

A total of 18 studies reporting iron [27, 31–35, 37, 39, 43, 45–49, 51, 52, 56, 58, 63] stated that SMD of SI in women who had GDM compared to pregnant women without GDM was 0.52 μg/L (95% CI: 0.18–0.85; *I*^2^ = 97.4%, *p* < 0.001) (Figure 5). SI levels were 0.52 μg/L higher in the GDM group compared to those in the non-GDM group, with a statistically significant difference.

### 3.5. Total Iron Binding Capacity

**Figure 6:**
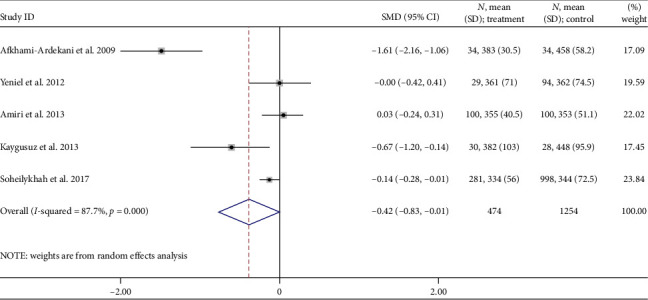
Mean TIBC concentration (μg/dL) differences.

Five studies examined TIBC during pregnancy and the risk of gestational diabetes [26, 30, 33, 38, 47]. Women who had GDM compared to pregnant women without GDM were −0.42 μg/dL (95% CI: −0.83 to −0.01; *I*^2^ = 87.7%, *p*=0.001) (Figure 6). The GDM group had a TIBC level 0.42 μg/dL lower than the non-GDM group, and the difference was statistically significant.

### 3.6. Transferrin

**Figure 7:**
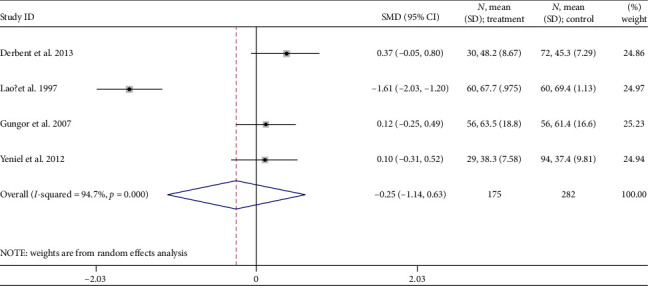
Mean transferrin concentration (μmol/L) differences.

Data pooled from four studies [36, 37, 47, 48] that compared GDM women with non-GDM groups showed no statistically significant difference in transferrin (TRF) levels −0.25 ng/mL (95% CI: −1.14 to 0.63; *I*^2^ = 94.7%, *p* < 0.001) (Figure 7).

### 3.7. Transferrin Receptor

**Figure 8:**
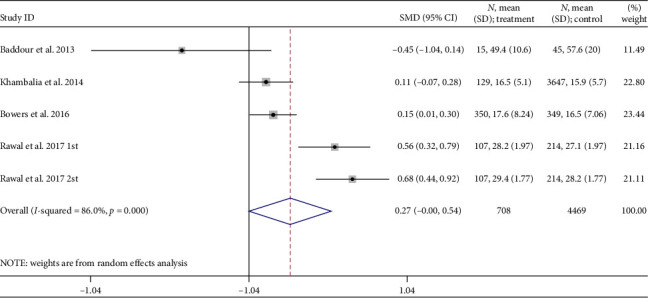
Transferrin receptor concentration (nmol/L) differences.

Four studies examined sTfR during pregnancy and the risk of GDM [24, 27, 28, 59]. The SMD of the transferrin receptor in women who had GDM compared to that in pregnant women without GDM was 0.27 nmol/L (95% CI: 0.00–0.54; *I*^2^ = 86%, *p* < 0.001) (Figure 8). The GDM group had a sTfR level 0.27 nmol/L higher than the non-GDM group, and the difference was statistically significant.

### 3.8. Dose-Responsive Meta-Analysis

**Figure 9:**
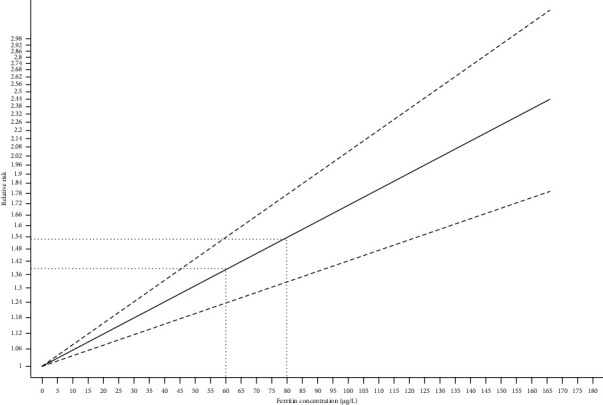
Linear dose–response analysis.

**Figure 10:**
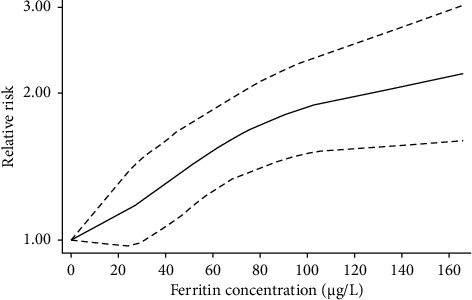
Nonlinear dose–response analysis.

Four studies [28, 40–42] were included to test the dose–response association. These studies reported three levels of RR for ferritin exposure. Nonetheless, this study was manifested by the dose–response testing for the quantitative assessment of the potential association between ferritin levels and GDM risks. The summary RR for an increment of 5 μg/L in ferritin levels was 2.71% in the risk of GDM (1.027 [95% CI: 1.018, 1.036]), Prob > chi^2^ = 0.2352. This value is greater than 0.05; thus, there is a linear relationship (Figure 9). To further confirm the relationship between the two, this study conducted a nonlinear analysis. A nonlinear dose–response analysis indicated that increased ferritin levels may lead to an elevated risk of developing gestational diabetes (Figure 10).

### 3.9. Subgroup Analysis

For the ferritin and iron levels and GDM risk, because many factors influence the development of GDM, this study performed subgroup analysis studies according to location, GDM diagnosis, trimester, study design, SF, assay age, BMI, parity, and sample size (Tables 3 and 4). Due to the low heterogeneity of the remaining iron metabolism indicators and the small number of included articles, subgroup analysis was not performed.

On stratification of meta-analysis by geographic location, the source of data was categorized as Asia, Africa, Occident, Middle East, and Europe. For SF, the SMD was 0.83 (0.23, 1.43) for African areas, 0.63 (0.18, 1.07) for Asia, and 2.84 (0.47, 5.22) for Occident. Occident and Africa had higher mean differences in the ferritin level development of GDM. Occident areas had a higher risk of GDM than African areas. For SI, the SMD was −0.20 (−0.41, 0.01) for Europe, 0.83 (0.19, 1.47) for Asia, and 0.32 (0.04, 0.60) for the Middle East. Asia areas had a slightly higher risk of GDM than Middle East areas.

When stratified using study design, in both case and cohort study groups, the SF level in the GDM group was significantly higher than that in the non-GDM group. For SF, the SMD of case–control studies 1.38 (0.51, 2.25) was higher than the cohort studies 1.08 (0.48, 1.67) development of GDM. For SI, the SMD of case–control studies 0.58 (−0.03, 1.19) was higher than the cohort studies 0.32 (0.05, 0.60) development of GDM. During retrospective case–control studies, there may be recall bias among participants and potential selection bias in the choice of controls, leading to inaccurate comparisons between patients and controls. Therefore, compared to cohort studies, retrospective case–control studies may show a higher SMD in SI and SF levels.

On stratification by study size, the SMD for SF was 0.38 (−0.24, 1.00) for *n* < 150, 1.07 (0.58, 1.55) for 150 ≤ *n* < 1000, and 4.15 (0.52, 7.78) for *n* ≥ 1000. For SI, the SMD was 0.50 (−0.06, 1.06) for *n* < 150, 0.77 (−0.20, 1.75) for 150 ≤ *n* < 1000, and 0.16 (−0.02, 0.34) for *n* ≥ 1000. Larger studies had higher mean differences in ferritin and iron level development of GDM.

This study stratified ferritin or iron measurement by trimester, categorizing them into first, second, and third trimesters. For SF, the SMD was 0.13 (−0.41, 0.66) for the first trimesters, 2.01 (0.61, 3.42) for the second trimesters, and 0.53 (0.13, 0.94) for the third trimesters. For SI, the SMD was 0.52 (−0.25, 1.29) for the first trimesters, 0.49 (0.12, 0.86) for the second trimesters, and 0.87 (−0.25, 2.00) for the third trimesters. The second trimester had the higher mean differences in ferritin and iron level development of GDM.

Stratifying the meta-analysis by ascertainment of GDM, the study was categorized as WHO, ADA, Medical Record, and IADPSG. For SF, the SMD was 0.53 (0.22, 0.84) for WHO, 1.26 (−0.01, 2.53) for ADA, 0.35 (0.18, 0.52) for Medical Record, and 1.98 (1.78, 2.17) for IADPSG. For SI, the SMD was 0.73 (0.04, 1.41) for WHO, 0.13 (−0.20, 0.45) for Medical Record, 0.47 (0.08, 0.87) for ADA, and 0.63 (−0.19, 1.44) for IADPSG. Ascertainment with WHO, Medical Record, IADPSG GDM group SF level is significantly higher than that of the non-GDM group, while the GDM group SI level is significantly higher than that of the non-GDM group in Ascertainment with WHO and ADA.

Stratifying the meta-analysis by SF assay, the review was categorized as MEIA, ELISA, IRMA, CLA, immunoturbidimetry, and NR (not reported). The SMD was 1.06 (0.03, 2.09) for MEIA, 3.08 (0.55, 5.61) for ELISA, 0.98 (0.48, 1.48) for IRMA, −0.13 (−0.88, 0.61) for CLA, 0.85 (−0.51, 2.21) for immunoturbidimetry, and 1.93 (1.69, 2.17) for NR.

The SF level in the GDM group was significantly higher than that in the non-GDM group, while there was no statistical difference between the two groups in other detection methods.

For SF and SI, this study conducted a subgroup analysis on potential confounding factors (BMI, age, parity) in the study subjects included in the included literature and found that these factors may affect the results of the meta-analysis.

### 3.10. Sensitivity Analysis

To further evaluate the stability of the research results, this study conducted a sensitivity analysis (Table 5). Sensitivity analysis is used to assess and validate the robustness and consistency of research results. By excluding or including specific studies, it evaluates their impact on the overall effect size. When certain articles exhibit significant methodological heterogeneity or bias, sensitivity analysis examines whether excluding these articles significantly alters the synthesized effect.

#### 3.10.1. SF Sensitivity Analysis

**Figure 11:**
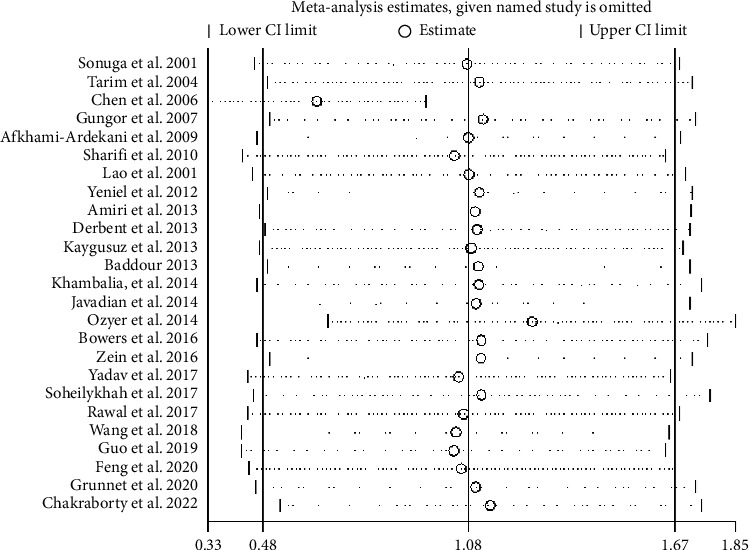
SF sensitivity analysis.

In order to further evaluate the stability of the research results, this study carried out a sensitive analysis. A sensitivity analysis of SF showed that Chakraborty 2022 [61], OZYER 2014 [39], and CHEN 2006 [35] studies are the main sources of heterogeneity (Figure 11). After excluding these 3 studies, the meta-analysis results of the remaining 22 studies were consistent with those before the exclusion, and the SI level in the GDM group was still higher than that in the non-GDM group (SMD = 0.86, 95% CI [0.58, 1.14], *p* < 0.001; *I*^2^ = 95.2%), indicating good stability of the results (Table 5).

#### 3.10.2. SI Sensitivity Analysis

**Figure 12:**
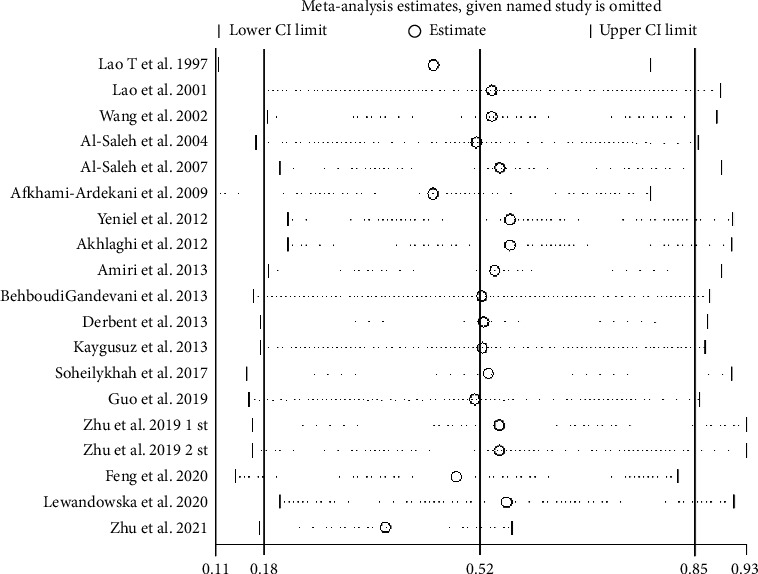
SI sensitivity analysis.

In the sensitivity analysis of iron (Figure 12), the results indicate that studies such as Lao 1997, Afkhami-Ardekani 2009, and Zhu 2021, among others, are the primary sources of heterogeneity. After excluding these three studies, the meta-analysis results of the remaining 16 studies remain consistent with those before exclusion. The analysis reveals that the levels of SF in the GDM group are still higher than those in the non-GDM group (SMD = 0.21, 95% CI [0.08, 0.38], *p* < 0.001; *I*^2^ = 84.1%), indicating good stability of the results.

#### 3.10.3. TIBC Sensitivity Analysis

**Figure 13:**
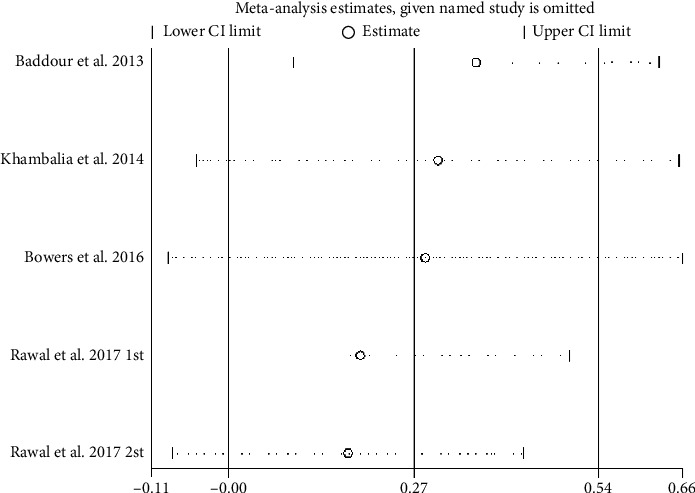
TIBC sensitivity analysis.

In the sensitivity analysis conducted for TIBC (Figure 13), the results indicate that Afkhami-Ardekani (2009) is a major source of heterogeneity. After excluding this literature, the meta-analysis results are inconsistent with those before exclusion (SMD = −0.13, 95% CI [−0.34, 0.07], *p* = 0.126; *I*^2^ = 47.5%). Compared to the non-GDM group, there is an increasing trend in TIBC in the GDM group, but the difference between the two groups is not statistically significant.

### 3.11. Publication Bias

In the meta-analysis comparing the differences in iron metabolism indicators between the GDM group and the non-GDM group, Begg's and Egger's tests were conducted for each iron metabolism indicator (Table 5). The results indicate that the *p*-values for both Begg's and Egger's tests for each indicator are greater than 0.05, suggesting a relatively low likelihood of publication bias.

## 4. Discussion

Diabetes is a life-threatening and costly condition, highlighting the importance of its prevention and treatment. This meta-analysis exhibits a positive correlation between high iron levels and GDM development. The current findings reveal that concentrations of ferritin, iron, transferrin receptor, and TIBC in patients with GDM were elevated than those in women without GDM.

Women with and without GDM did not show significant differences in TIBC and transferrin concentration, so their higher levels may help to diagnose GDM during the first and second trimesters of pregnancy. It indicates whether pregnant women need medical intervention. Therefore, women's clinical health urges to assess body iron stores. Iron level is truly determined by none of the single circulating iron biomarkers. Iron, ferritin, transferrin, saturated transferrin, and soluble transferrin receptors exhibit promising clinical effects when administered in combinations. Nonetheless, none of the included studies determined all iron biomarkers, while most of the studies tested ferritin levels as an iron biomarker for the investigation of GDM. Therefore, the present study is an attempt to find representative indicators of iron storage and its association with GDM.

The pathophysiology of GDM is still vague. This study reports a positive association of high SF with GDM. The ferritin results are consistent with previous meta-analyses [64–66]. SF protein is known to store iron, so the SF level is a sensitive and suitable indicator for the estimation of body iron stores that indicate iron deficiency or iron overload by detecting SF levels [67, 68]. Researchers hypothesized that the maternal iron status overload promotes ROS levels and oxidative stress, and pregnant women are more sensitive to oxidative stress, which may be tightly linked processes to cellular injury and apoptosis of β-cells and increases insulin resistance, and likewise, ferritin as a redox-active transitional metal may induce a modification in certain inflammatory processes [33, 69–74].

Since macrophages sequester iron, infections and inflammatory conditions are often associated with a decrease in the SI level. Alternatively, iron payload leads to increased levels of SI. Free iron is extremely poisonous and plays a critical role in oxidative stress and free radical generation [71]. SI concentration tested immediately after the administration of iron supplements is always high, so this factor must be considered for the accurate determination of SI level. Therefore, measuring iron alone does not accurately reflect body iron reserves [75].

In this study, the serum TIBC was lower in the GDM group than in the non-GDM group. TIBC is of great significance in the diagnosis of iron deficiency anemia and iron overload. Iron binding potential is the ability of transferrin to bind to iron. When the body is overloaded with iron, the content of free transferrin in the blood is reduced, resulting in a lower TIBC value.

The concentration of sTfR also points out the iron level in the presence of iron deficiency, and serum sTfR increases [76]. Inflammatory cytokines enhance transferrin receptors, promoting iron accumulation in tissues [77]. In women with inflammatory or infectious disorders, plasma ferritin levels can be falsely elevated. To minimize certain potential confounding factors, studies have proposed the use of soluble transferrin receptors (sTfR) to assess iron levels in inflammatory conditions instead of SF. However, sTfR's contribution to GDM development is debatable, as indicated by the cited references. Some studies support this perspective [27, 37, 78], while others present opposing views [28, 79].

This meta-analysis revealed a considerable association of GDM risk with neither transferrin receptors nor serum transferrin levels. Moreover, this meta-analysis exhibited some heterogeneity in the findings of certain studies, and this study conducted subgroup analyses for SI and ferritin. The findings suggest that regional differences, gestational period of blood collection, sample size, study design, ascertainment of GDM, and the method used for the SF assay could be potential sources of heterogeneity.

The data showed that adjusting BMI, family history of diabetes, and advanced maternal age have a significant impact on heterogeneity. Our findings are consistent with the previous results [80] study that the most frequently reported determinants of GDM were maternal obesity, family history of diabetes, and advanced maternal age.

This study encountered several limitations. Firstly, the effect of inflammatory biomarkers on GDM was not assessed. No association has been detected between GDM risk and iron supplement intake. High SF levels were linked with an elevated risk of GDM development compared to low levels of SF. This study did not assess a relationship between the defined threshold of augmented levels and GDM. At the same time, the number of studies was inadequate for a meta-analysis of the SI index. On the other hand, certain aspects impart the strength to this study. Our study is significant as the body iron status may serve as a novel biomarker for the prevention of GDM risk. However, these findings must be confirmed by executing it prospectively on a large scale. The possible outcomes will help to investigate the underlying physiological mechanisms. Further studies are needed to evaluate both novel and traditional iron biomarkers during the preconception period in women planning pregnancy, particularly those with a history of GDM.

## 5. Conclusion

In summary, this meta-analysis suggests an association of elevated ferritin with high iron concentrations and low TIBC with an increased risk of gestational diabetes (GDM). Specifically, this study exhibited a significant association between higher levels of ferritin and a greater possibility of GDM development. However, this conclusion does not offer definitive evidence establishing a relationship among serum transferrin, sTfR, and GDM. As a practical implication, assessing the concentrations of iron, ferritin, or TIBC in early pregnancy could serve as a valuable tool for identifying pregnant women at a risk of developing GDM. However, the clinical applicability of these findings remains limited due to the absence of specific cutoff values for ferritin, iron, and TIBC that could guide risk assessment in clinical settings. Future studies should focus on defining clinically relevant thresholds that can aid in decision-making regarding screening and early intervention. However, this study did not systematically evaluate the specificity and sensitivity of these biomarkers in predicting GDM risk. Further research is needed to validate these findings across diverse populations and assess the potential influence of factors such as ethnicity, dietary habits, and genetic predisposition on iron metabolism and GDM risk. Furthermore, in the mechanism linking iron and diabetes, there are several important chemical substances and compounds not studied in this paper. These include iron reductases, oxidoreductases (such as hydrogen peroxide and superoxide dismutase), iron carriers, complexes (such as iron bound to hemoglobin, iron–sulfur complexes), reactive oxygen species, and others. These compounds may affect insulin sensitivity, insulin secretion, and glucose metabolism through various pathways, influencing the onset and development of GDM. Therefore, further prospective cohort studies are needed to establish the relationship between iron-related compounds and the occurrence of GDM.

## Figures and Tables

**Table 1:** Characteristics of the 39 studies included in the quantitative analysis.

Authors year	Location (ethnicity)	Study design	Participant age (mean or ranges provided)	Participant	GDM size	Trimester for biomarker collection	Ascertainment of GDM	SI	SF	sTfR	TIBC	TRF	Serum Ferritin assay	Matching/variables adjustment
Khambalia et al. 2014 [24]	Australia	Cohort	NR	3776	129	First trimester	Medical record		※	※			ELISA	Age, country of birth, parity, maternal weight, smoking during pregnancy, hypertensive disorders
Yadav et al. 2017 [25]	India	Case–control	NR	90	30	Second trimester	Carpenter and Coustan		※				ELISA	None
Soheilykhah et al. 2017 [26]	Iran	Cohort	NR	1279	281	First trimester	WHO	※	※		※		ELISA	Age, BMI
Bowers et al. 2016 [27]	Denmark	Cohort	25.7–36.5	699	350	First trimester	WHO		※	※			Immunoturbidimetry	Adjusted for age, FHD, ever exercise in pregnancy, parity (only in sTfR models), BMI, CRP, and oxidized LDL
Rawal et al. 2017 [28]	USA	Case–control	18–40	321	107	First/second trimester	Carpenter and Coustan		※	※			ELISA	Maternal age, gestational age at blood collection, nulliparity, education, FHD, BMI and CRP
Zein et al. 2015 [29]	Lebanon	Cohort	20–33	104	16	First trimester	IADPSG		※				NR	None
Amiri et al. 2013 [30]	Iran	Case–control	NR	200	100	Second trimester	ADA	※	※		※		IRMA	Age, parity, body mass index, gestational age
Wang et al.2013 [31]	Iran	Cohort	27.57 ± 4.84	1033	72	Second trimester	ADA	※						Age, BMI, educational level, number of pregnancies, passive smoking, FHD, serum zinc/iron and hemoglobin levels, and deficient zinc/iron intakes in early pregnancy
Javadian et al. 2014 [32]	Iran	Case–control	22–38	102	52	Second trimester	ADA		※				IRMA	Duration of pregnancy; age
Afkhami-Ardekani and Rashidi 2009 [33]	Iran	Case–control	NR	68	34	Second trimester	ADA	※	※		※		IRMA	Age; BMI; parity
Feng et al. 2020 [34]	China	Case–control	25–36	150	75	Second trimester	ADA	※	※				Immunoturbidimetry	Age
Chen et al. 2006 [35]	USA	Cohort	22.1 ± 0.7	1456	45	Second trimester	Carpenter and Coustan		※				ELISA	Age, ethnicity, parity, FHD, gestational age at blood collection, and cigarette smoking, BMI
Derbent et al. 2013 [36]	Turkey	Case–control	23–37	102	30	Second trimester	ADA	※	※			※	CLA	Age; BMI
Gungor et al. 2007 [37]	Turkey	Case–control	21–34	112	56	Third trimester	ADA		※			※	MEIA	Age; BMI
Kaygusuz et al. 2013 [38]	Turkey	Case–control	22–33	58	30	Second trimester	Carpenter and Coustan	※	※		※		CLA	Maternal age, gravidity, maternal pre-conceptional BMI, BMI
Ozyer et al. 2014 [39]	Turkey	Case–control	25–36	105	35	First trimester	ADA		※				CLA	BMI
Sharifi et al. 2010 [43]	Iran	Case–control	25–35	128	64	Second trimester	ADA		※				IRMA	C-reactive protein, BMI, blood pressure, history of GDM and FHD
Soubasi et al. 2010 [44]	Greece	Cohort	24–37	69	6	Third trimester	ADA		※				ELISA	None
Lao et al. 2001 [45]	China	Case–control	NR	291	97	Third trimester	WHO	※	※				MEIA	Parity; age; BMI
Tarim et al. 2004 [46]	Turkey	Cohort	21.8–31.4	253	20	Second trimester	ADA		※				IRMA	None
Yeniel et al. 2012 [47]	Turkey	Case–control	NR	123	29	First trimester	ADA	※	※		※	※	CLA	Age, BMI, gravidity and parity
Lao and Tam 1997 [48]	China	Cohort	NR	120	60	Third trimester	WHO	※				※		None
Akhlaghi et al. 2012 [49]	Iran	Case–control	NR	60	30	Second trimester	ADA	※						None
Al-Saleh et al. 2004 [50]	Kuwait	Case–control	23–34	30	15	Third trimester	ADA	※						None
Guo et al. 2019 [51]	China	Case–control	NR	362	52	First trimester	IADPSG	※	※				MEIA	None
Cheng et al. 2020 [40]	China	Cohort	30.16 ± 3.31	851	132	First/second trimester	IADPSG		※				CLA	None
Lewandowska et al. 2020 [52]	Poland	Cohort	18–45	563	110	First trimester	IADPSG	※						None
Zhu et al. 2021 [53]	China	Case–control	NR	610	305	First trimester	IADPSG	※						Gestational age, prepregnancy BMI, parity, annual household income, education level, and passive smoking, plasma levels of magnesium, zinc, calcium, copper, selenium, and chromium
Zein et al. 2016 [54]	Lebann	Cohort	NR	93	79	First trimester	WHO		※				CLA	None
Zhang et al. 2021 [41]	China	Cohort	18∼	2117	219	Second trimester	IADPSG		※				ELISA	Maternal age, BMI, years of education, monthly average income per capita, nulliparous, FHD, during pregnancy). Gestational week of blood sampling
Sonuga and Sonuga 2001 [55]	Nigeria	Case–control	18–45	80	40	Second trimester	WHO		※				ELISA	Age
Zhu et al. 2019 [56]	China	Cohort	26.4 ± 3.7	3289	429	First/second trimester	IADPSG	※						Age, pre-pregnancy BMI, FHD, parity, smoking, drinking, diastolic blood pressure, systolic blood pressure, gestational week at visit, income, and education, arsenic, copper, and selenium
Wang et al. 2018 [57]	China	Case–control	20–40	739	701	Second trimester	WHO and IADPSG		※				NR	None
Casanueva and Viteri 2003 [58]	China	Case–control	NR	136	49	NR	NR	※						None
Baddour et al. 2013 [59]	Canada	Case–control	29.1 ± 4.8	60	15	Third trimester	Medical record		※	※			CLA	None
Grunnet et al. 2020 [60]	Tanzania	Case–control	392	392	153	Third trimester	WHO		※				CLA	Age and BMI
Shareef et al. 2022 [61]	India	Cohort	NR	80	12	First trimester	ADA		※				CLA	None
Yang et al. 2022 [42]	China	Cohort	29–35	2327	534	Second trimester	IADPSG		※				CLA	Age, BMI, education background, smoking status, alcohol consumption status, conception method, parity, abortion history, thalassemia history and hemoglobin
Al-Saleh et al. 2007 [62]	Kuwait	Case–control	18–45	21	10	NR	Medical record	※						None

Abbreviations: ADA = American diabetes association, BMI = body mass index, Carpenter and Coustan = Carpenter and Coustan diagnostic criteria, CLA = chemiluminescence assay, CRP = C reactive protein, ELISA = enzyme-linked immuno sorbent assay, FHD = family history of diabetes, IADPSG = international association of diabetes and pregnancy study groups, IRMA = immuno radio metric assay, OGTT = oral glucose tolerance test, SD = standard deviation, T = tertiles, WHO = World Health Organization.

^※^The sign represents the indicator studied in this article.

**Table 2:** Quality evaluation of the studies with Newcastle–Ottawa.

**Case–control studies**	**Selection**				**Comparability**		**Exposure**			**Quality score**
**Author**	**Case definition adequate**	**Representativeness of the cases**	**Selection of controls**	**Definition of controls**	**Study controls for age**	**Study controls for other factors**	**Ascertainment of exposure**	**Same method of ascertainment for cases and controls**	**Non-response rate**	

Yadav et al. 2017	1	1	1	1	0	0	1	1	0	6
Rawal et al. 2017	1	1	1	1	1	1	1	1	0	8
Amiri et al. 2013	1	1	1	1	1	1	1	1	0	8
Javadian et al. 2014	1	1	1	1	1	0	1	1	0	7
Afkhami-Ardekani et al. 2009	1	1	1	1	1	1	1	1	0	8
Feng et al. 2020	1	1	1	1	1	0	1	1	0	7
Derbent et al. 2013	1	1	1	1	1	1	1	1	0	8
Gungor et al. 2007	1	1	1	1	1	1	1	1	0	8
Kaygusuz et al. 2013	1	1	1	1	1	1	1	1	0	8
Ozyer et al. 2014	1	1	1	1	0	1	1	1	0	7
Sharifi et al. 2010	1	1	1	1	0	1	1	1	0	7
Lao et al. 2001	1	1	1	1	1	1	1	1	0	8
Yeniel et al. 2012	1	1	1	1	1	1	1	1	0	8
Akhlaghi et al. 2012	1	1	1	1	0	0	1	1	0	6
Al-Saleh et al. 2004	1	1	1	1	0	0	1	1	0	6
Guo et al. 2019	1	1	1	1	0	0	1	1	0	6
Zhu et al. 2021	1	1	1	1	0	0	1	1	0	6
Sonuga et al. 2001	1	1	1	1	1	0	1	1	0	7
Wang et al. 2018	1	1	1	1	0	0	1	1	0	6
Wang et al. 2002	1	1	1	1	0	0	1	1	0	6
Baddour et al. 2013	1	1	1	1	0	0	1	1	0	6
Grunnet et al. 2020	1	1	1	1	1	1	1	1	0	7
Al-Saleh et al. 2007	1	1	1	1	0	0	1	1	0	6

**Cohort studies**	**Selection**				**Comparability**		**Outcome**			**Quality score**
**Author**	**Representativeness of exposed cohort**	**Selection of the non-exposed cohort**	**Ascertainment of exposure**	**Outcome of interest was not present**	**Study controls for age**	**Study controls for other factors**	**Ascertainment of outcome**	**Follow-up enough for outcome**	**Adequacy of follow-up**	

Khambalia et al. 2014	1	1	1	1	1	1	1	1	0	8
Soheilykhah et al. 2017	1	1	1	1	1	1	1	1	1	9
Bowers et al. 2016	1	1	1	1	1	1	1	0	1	8
Zein et al. 2015	1	1	1	1	1	1	1	1	0	8
Behboudi-Gandevani et al. 2013	1	1	1	1	1	1	1	1	1	9
Chen et al. 2006	1	1	1	1	1	1	1	1	1	9
Soubasi et al. 2010	1	1	1	1	0	0	1	1	1	7
Tarim et al. 2004	1	1	1	1	0	0	1	1	1	7
Lao et al. 1997	1	1	1	1	0	0	1	1	1	7
Cheng et al. 2020	1	1	1	1	0	0	1	1	1	7
Lewandowska et al. 2020	1	1	1	1	0	0	1	1	1	7
Zein et al. 2016	1	1	1	1	1	1	1	1	0	8
Zhang et al. 2021	1	1	1	1	1	1	1	1	1	9
Zhu et al. 2019	1	1	1	1	1	1	1	1	1	9
Chakraborty et al. 2022	1	1	1	1	0	0	1	1	1	7
Yang et al. 2022	1	1	1	1	1	1	1	1	1	9

**Table 3:** Subgroup analysis of the risk of GDM and ferritin.

Subgroup		No. of studies	SMD (95% CI)	*I* ^2^ (%)	*P*′	*P* ^″^
Region	Occident	5	2.84 (0.47, 5.22)	99.8	0.001	0.019
	Africa	2	0.83 (0.23, 1.43)	81.5	0.020	0.006
	Asia	18	0.63 (0.18, 1.07)	96.7	0.001	0.006

Study design	Case–control	19	1.38 (0.51, 2.25)	99.1	0.001	0.002
	Cohort	6	1.08 (0.48, 1.67)	39.4	0.143	0.002

Sample size	*n* < 150	13	0.38 (−0.24, 1.00)	95.4	0.001	0.226
	150 ≤ *n* < 1000	9	1.07 (0.58, 1.55)	97.0	0.001	0.001
	*n* ≥ 1000	3	4.15 (0.52, 7.78)	99.9	0.001	0.025

Trimester	T1	9	0.13 (−0.41, 0.66)	97.6	0.001	0.644
	T2	12	2.01 (0.61, 3.42)	99.3	0.001	0.005
	T3	4	0.53 (0.13, 0.94)	84.6	0.001	0.01

Ascertainment of GDM	WHO	6	0.53 (0.22, 0.84)	91.1	0.001	0.001
	ADA	15	1.26 (−0.01, 2.53)	99.3	0.001	0.052
	Medical record	2	0.35 (0.18, 0.52)	0.0	0.951	0.001
	IADPSG	2	1.98 (1.78, 2.17)	0.0	0.496	0.001

Serum Ferritin assay	MEIA	3	1.06 (0.03, 2.09)	96.8	0.001	0.044
	ELISA	5	3.08 (0.55, 5.61)	99.8	0.001	0.017
	IRMA	6	0.98 (0.48, 1.48)	91.0	0.001	0
	CLA	8	−0.13 (−0.88, 0.61)	95.5	0.001	0.725
	Immunoturbidimetry	2	0.85 (−0.51, 2.21)	97.9	0.001	0.221
	NR	1	1.93 (1.69, 2.17)	—	—	0

Adjust for age	Adjusted	16	1.38 (0.62, 2.13)	99.2	0.001	0.001
	Unadjusted	9	0.54 (−0.44, 1.51)	97.8	0.001	0.279

Adjust for BMI	Adjusted	14	1.23 (0.29, 2.17)	99.4	0.001	0.010
	Unadjusted	11	0.90 (0.38, 1.41)	95.5	0.001	0.001

Adjust for parity	Adjusted	8	2.10 (0.61, 3.58)	99.6	0.001	0.006
	Unadjusted	17	0.61 (0.14, 1.08)	96.9	0.001	0.011

*Note:P*′ = *p* value of heterogeneity test, *P*^″^ = *p* value of significance.

**Table 4:** Subgroup analysis in the risk of GDM and iron.

Subgroup		No. of studies	SMD (95% CI)	*I* ^2^ (%)	*P*′	*P* ^″^
Region	Asia	8	0.83 (0.19, 1.47)	98.9	0.001	0.011
	Middle East	10	0.32 (0.04, 0.60)	82.7	0.001	0.024
	Europe	1	−0.20 (−0.41, 0.01)	—	—	0.063

Sample size	*n* < 150	9	0.50 (−0.06, 1.06)	91.3	0.001	0.082
	150 ≤ *n* < 1000	6	0.77 (−0.20, 1.75)	98.8	0.001	0.120
	*n* ≥ 1000	4	0.16 (−0.02, 0.34)	87.5	0.001	0.089

Study design	Cohort	6	0.32 (0.05, 0.60)	94.7	0.001	0.022
	Case–control	13	0.58 (−0.03, 1.19)	97.2	0.001	0.061

Ascertainment of GDM	WHO	3	0.73 (0.04, 1.41)	95.8	0.001	0.037
	Standard medical record	2	0.13 (−0.20, 0.45)	0.0	0.386	0.455
	ADA	9	0.47 (0.08, 0.87)	88.6	0.001	0.019
	IADPSG	5	0.63 (−0.19, 1.44)	99.3	0.001	0.130

Trimester	T1	6	0.52 (−0.25, 1.29)	99.1	0.001	0.187
	T2	8	0.49 (0.12, 0.86)	92.2	0.001	0.010
	T3	3	0.87 (−0.25, 2.00)	95.2	0.001	0.128
	NR	2	0.13 (−0.20, 0.45)	0.0	0.386	0.455

Adjust for age	Adjusted	11	0.65 (0.08, 1.22)	98.0	0.001	0.026
	Unadjusted	8	0.34 (0.03, 0.65)	92.1	0.001	0.032

Adjust for BMI	Adjusted	13	0.38 (−0.03, 0.80)	98.0	0.001	0.071
	Unadjusted	6	0.81 (0.37, 1.24)	88.8	0.001	0.001

Adjust for parity	Adjusted	11	0.65 (0.08, 1.22)	97.4	0.001	0.026
	Unadjusted	8	0.34 (0.03, 0.65)	92.1	0.001	0.032

*Note:P*′ = *p* value of heterogeneity test, *P*^″^ = *p* value of significance.

**Table 5:** Heterogeneity and publication bias and sensitivity analysis.

		Number of studies	SMD (95% CI)	Heterogeneity		Significance of effect		Bias test	
				*I* ^2^ (%)	*P*	*z*	*p*	Begg's	Egger's
SF	a	25	1.08 (0.48, 1.67)	99	0.001	3.56	0.001	0.981	0.196
	b	22	0.86 (0.58, 1.14)	95.2	0.001	6.01	0.001	0.693	0.053

SI	a	19	0.52 (0.18, 0.85)	97.4	0.001	3.03	0.002	0.184	0.187
	b	16	0.21 (0.08, 0.38)	84.1	0.001	2.68	0.007	0.499	0.209

TIBC	a	5	−0.42 (−0.83, −0.01)	87.7	0.001	1.98	0.047	0.086	0.326
	b	4	−0.13 (−0.34, 0.07)	47.5	0.126	1.28	0.199	0.734	0.791

## Data Availability

All the data have been incorporated to this article.
